# Learning Anatomy: Can Dissection and Peer-Mediated Teaching Offer Added Benefits over Prosection Alone?

**DOI:** 10.5402/2013/873825

**Published:** 2013-03-28

**Authors:** Lynn Ashdown, Evan Lewis, Maxwell Hincke, Alireza Jalali

**Affiliations:** Faculty of Medicine, University of Ottawa, 451 Smyth Road, Ottawa, ON, Canada K1H 8M5

## Abstract

*Purpose*. To evaluate the impact of an optional thoracic dissection elective upon anatomy subject acquisition and determine whether peer-mediated teaching has a beneficial effect. *Methodology*. First year medical students' results on thoracic anatomy laboratory examinations over a five-year period were obtained. All students were taught in the laboratory using prosected specimens as part of a standard curriculum. A subset of students from each class volunteered to participate in an optional thoracic dissection. A comparison of exam performance between the two groups was made, and the results were analyzed to see if incorporating peer teaching into the elective had an impact on the students' performance on anatomy examinations. *Results*. With the exception of one year's results, no significant statistical difference was found in student performance on anatomy examinations between the two groups. The addition of peer teaching did not result in superior performance. *Conclusion*. It is believed that prosected specimens are suitable for anatomy laboratory teaching in an undergraduate medical curriculum. Our study did not reveal that an opportunity for dissection offered any added benefit in terms of exam performance. In addition, peer teaching did not affect exam performance. This study strictly compared student exam results. It did not assess the possible impact of the dissection process to influence student attitudes towards death or the development of clinically relevant visuospatial abilities and procedural skills.

## 1. Introduction

With more schools utilizing prosected cadaveric specimens in the anatomy laboratory, is there still a role for dissection? Historically, dissection has been utilized as the best means for teaching anatomy to medical students [1]. Nevertheless, a systematic literature review found little objective data that actually supports this notion [2]. More recently, the superiority of dissection over prosection has been debated [3], and the use of prosection as an adequate tool for learning anatomy is becoming increasingly common in medical schools. A further understanding of the usefulness of both the aforementioned techniques is important as we look to the future and attempt to optimize gross anatomy education. Prior to commencing this study, our hypothesis was that first-year medical students who participate in an extracurricular thoracic dissection elective would perform better on thoracic-related anatomy examinations than those who did not. A secondary hypothesis was that the use of peer-mediated teaching would offer an added benefit to medical students learning gross anatomy. The aim of this study then was to evaluate the impact of an optional thoracic dissection elective upon anatomy subject acquisition and determine whether peer-mediated teaching has a beneficial effect. 

## 2. Method

A retrospective cohort study was performed. All first-year medical students (*n* = 709) at the University of Ottawa between 2002 and 2006 were taught thoracic anatomy in the cadaver lab through the use of prosected specimens as part of the standard curriculum. An optional thoracic anatomy dissection elective was offered to all students in addition to the regular curriculum. The dissection elective consisted of 12 hours in the cadaver lab, divided into 4 sessions. The focus of this elective was the dissection of specimens and a review of thoracic anatomy learned in class. 

**Figure 1:**
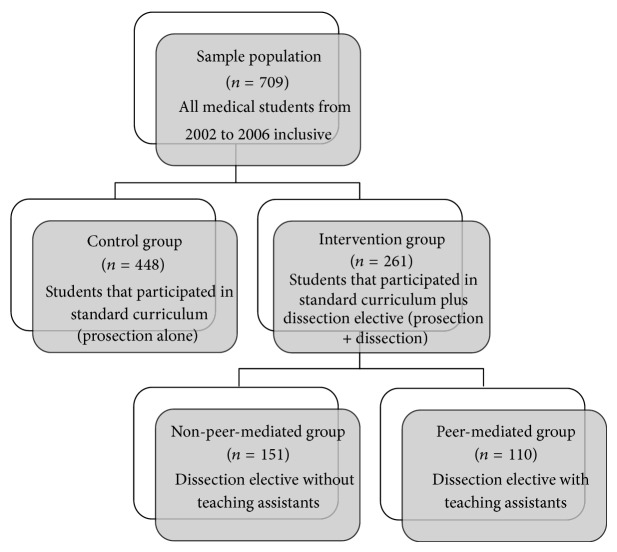
Distribution of sample population.

Figure 1 outlines how the sample population was distributed for analysis. Two groups were separated: the control group comprising the students who only participated in the standard curriculum; and the intervention group comprising the students who participated in the standard curriculum, and the dissection elective. Evaluation of the intervention was performed by analyzing students' results on the thoracic anatomy component of their examinations during the first year cardiology and respirology blocks. Examination scores were compared and analyzed using a paired Student's *t*-test.

A secondary analysis compared exam results between students in the dissection elective who received peer-mediated teaching and those who did not (Figure 1). From 2002 to 2004, there were no teaching assistants and the dissection elective was self-directed with the anatomy professor present to answer questions and demonstrate proper dissection technique. From 2005 to 2006, the role of the anatomy professor remained the same but senior medical students in their second year of study, who had taken the dissection elective previously, were also present as peer-mediators. Examination results between these two groups were analyzed using a paired Student's *t*-test.

## 3. Results

**Figure 2:**
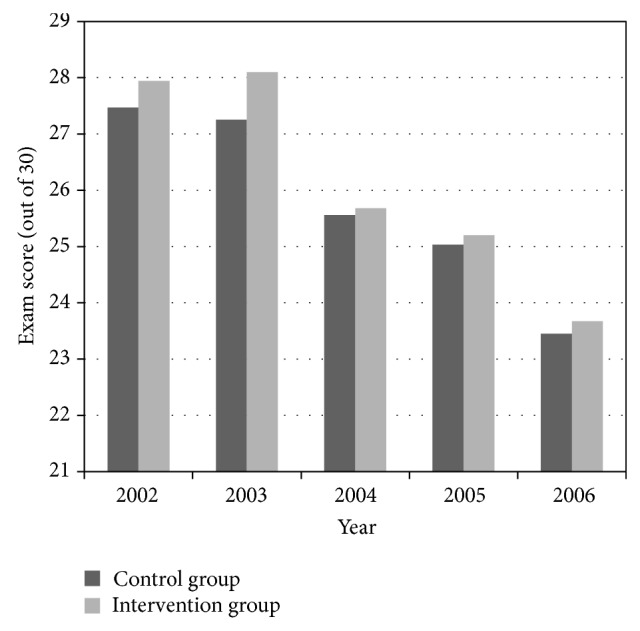
Comparison of exam results between control and intervention group: cardiology exam. Results of Cardiac Thoracic Anatomy Examination: control versus intervention groups.

**Figure 3:**
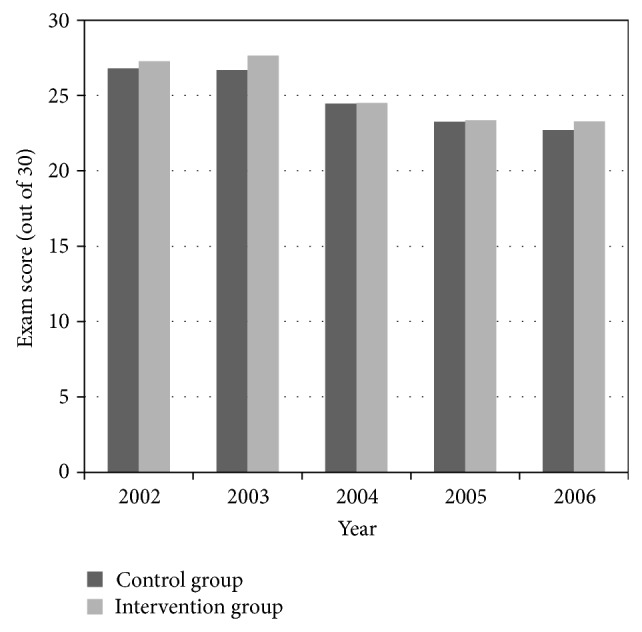
Comparison of exam results between control and intervention groups: respirology exam. Results of Respirology Thoracic Anatomy Examination: control versus intervention groups.

Comparison of scores on the thoracic anatomy components of the cardiology and respirology examinations between the control and intervention groups is depicted in Figures 2 and 3. Students' mean performances on the exams were consistently higher in the intervention group. However, with the exception of 2003 (*P* = 0.000979 and *P* = 0.00113 for cardiology and respirology exams, resp.), no statistical significance was found when mean scores were compared.

**Figure 4:**
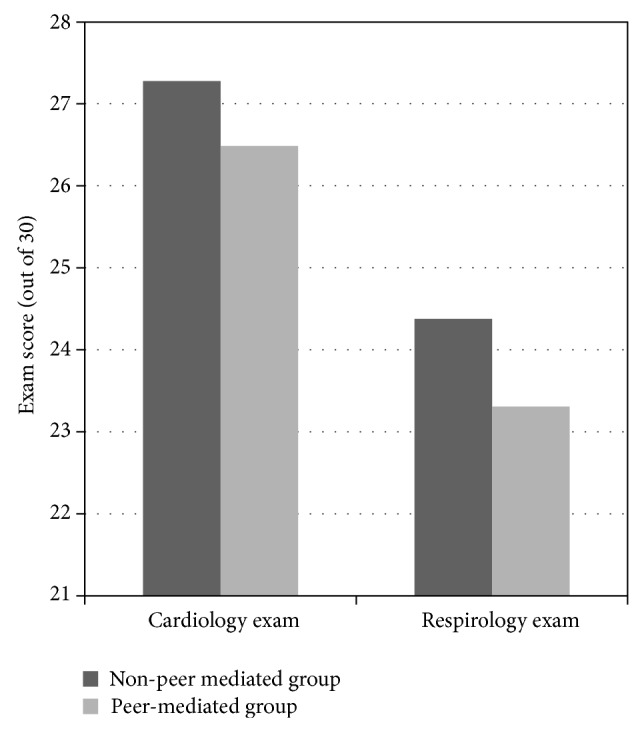
Comparison of exam results between students that did and did not receive peer-mediated teaching during the dissection elective.

Figure 4 compares performance on the thoracic anatomy component of the first-year cardiology and respirology exams between the Non-Peer-Mediated group and Peer-Mediated group. Student's *t*-test showed that the Non-Peer-Mediated group performed better on both examinations (*P* = 0.0033); however no statistical significance was elicited.

## 4. Discussion

Historically, dissection has been the gold standard for teaching anatomy to medical students [1], even though there is a dearth of objective data that does not support this notion [2]. Numerous studies have suggested that prosection is just as efficient as dissection for students learning gross anatomy [3, 4]. Our study aimed to identify whether introducing dissection of cadavers into the curriculum would offer any added educational benefit over students viewing prepared prosected specimens alone. Analysis of our data did suggest that students exposed to dissection above and beyond the regular curriculum of prosection alone performed slightly better on their anatomy examinations of the thorax compared with those students who did not have the additional benefit of dissection. It is important to note, however, that no statistical significance was identified between students learning gross anatomy from prosection alone and those students who had additional exposure to dissection in the anatomy lab. This is in keeping with the literature that prosection is just as efficacious as dissection for learning gross anatomy. As far back as 1990, Nnodim [5] showed that prosection was advantageous because it is more cost-effective and requires less time to learn the same amount of material. In fact, in a time when funding is scarce, showing that prosection is as adequate as dissection for acquisition of anatomy knowledge in medical school is important. 

It should be noted though that this study only analyzed dissection and prosection in terms of the acquisition and recall of thoracic anatomy knowledge. Literature suggests that dissection appears to offer benefits to students concerning their attitudes towards death and the development of clinically relevant visuospatial abilities and procedural skills [6]. Considering the retrospective nature of our study, we were unable to assess these variables. This study, therefore, could lay the groundwork for future investigations into the added benefits of dissection beyond acquisition of anatomical knowledge.

A second arm of this study was to determine whether the addition of peer-mediated teaching would offer an additional learning benefit to students participating in the dissection elective. Our study suggests that the addition of peer-mediated teaching to the dissection elective did not influence student performance on examinations. In fact, a surprising result suggested that the addition of teaching assistants may have negatively impacted student performance. Based on this interesting and unpredicted result, further exploration into this subject is warranted. Worthy of note, however, is that a study by Krych et al. [7] suggests that peer teaching improves anatomy subject acquisition and communication skills for the peer-teachers themselves. This was not examined in our study and may be an area that warrants further exploration. 

Although our study presents promising and useful data that prosection is a sufficient means by which to teach medical students gross anatomy, a number of limitations did exist within the study. Only quantitative data was collected. The additional benefit of qualitative data from medical students via focus groups or questionnaires would likely have been of benefit and perhaps would have added strength and another dimension to the study. Furthermore, this study is retrospective in nature. Future comparative studies could offer more information from a prospective study approach.

## 5. Conclusion

A greater understanding of pedagogical techniques is always an area of interest in medical education, and this study aimed to evaluate two of those techniques. First, our study suggests that prosection is as effective as dissection for acquisition of thoracic anatomy knowledge. This lays the groundwork for larger, more extensive studies to examine this notion further. Second, peer-mediated teaching appeared to offer no additional benefit with regards to anatomy knowledge acquisition. Considering the potential secondary benefit of creating peer-mediated teaching curricula, further exploration in this area through prospective, controlled studies is warranted.
